# Persistent calcium dependence after total thyroidectomy: a hypothesis of bone hunger syndrome with parathyroid reserve insufficiency in the setting of normalized parathyroid hormone - a case report and literature review

**DOI:** 10.3389/fendo.2026.1901308

**Published:** 2026-07-24

**Authors:** Wei Sun, Jian Wang

**Affiliations:** Department of Thyroid and Breast Surgery, Ma’anshan People’s Hospital, Ma’anshan, Anhui, China

**Keywords:** bone hunger syndrome, hypoparathyroidism, parathyroid hormone, thyroidectomy, vitamin D deficiency

## Abstract

**Background:**

Restoration of parathyroid hormone (PTH) to the normal range following total thyroidectomy is conventionally regarded as an indicator of functional recovery of the parathyroid glands. Nevertheless, a subset of patients develop hypocalcemia upon withdrawal of supplementation. This report describes a case in which PTH normalized postoperatively yet calcium supplements could not be discontinued for three years, and discusses the underlying mechanisms and management strategy.

**Case summary:**

A 40-year-old woman with multifocal papillary thyroid carcinoma harboring the BRAF V600E mutation underwent total thyroidectomy with bilateral level VI lymph node dissection. The patient had a background of Hashimoto’s thyroiditis (thyroglobulin antibody 225.30 IU/mL, thyroid peroxidase antibody 144.40 IU/mL). Preoperative serum calcium and PTH were within normal limits, but 25-hydroxyvitamin D was severely deficient (12.8 ng/mL). On postoperative day 1, hypocalcemic symptoms accompanied by a decline in serum calcium occurred and resolved after intravenous calcium administration. PTH transiently rose to 70.8 pg/mL on postoperative day 3 but fell to 3.0 pg/mL by one month after surgery. At three months postoperatively, PTH recovered to 26.7 pg/mL and remained consistently normal over the subsequent three years. However, repeated attempts to taper or discontinue calcium and active vitamin D at 3, 5, and 18 months postoperatively, as well as on multiple later occasions-provoked numbness of the hands and perioral region within 4–7 days. Emergency laboratory evaluations consistently revealed decreased serum calcium without a compensatory rise in PTH. At three years after surgery, the patient still depends on oral calcium supplementation to maintain normocalcemia.

**Conclusion:**

Normal basal PTH levels do not exclude parathyroid reserve insufficiency. In this patient, preoperative vitamin D deficiency may have predisposed to an osteomalacic state, potentially generating a persistent skeletal calcium demand when combined with a limited PTH stress response. We hypothesize that this represents a phenotype of ‘bone hunger syndrome with parathyroid reserve insufficiency’. Such patients require long-term individualized calcium replacement, and assessment of the calcium stress response facilitates precise phenotyping. However, further studies with objective biomarkers are needed to validate this hypothesis.

## Introduction

Postoperative hypocalcemia is one of the most common complications following total thyroidectomy, with a reported incidence of 7-51% (1, 2). The principal causes include intraoperative injury to the parathyroid glands, disruption of their blood supply, or inadvertent gland excision, leading to transient or permanent hypoparathyroidism (3, 4). Patients who remain dependent on calcium and active vitamin D supplementation to maintain normocalcemia at six months postoperatively are generally classified as having permanent hypoparathyroidism (5). In those with reversible hypoparathyroidism, PTH levels typically return to normal within weeks to six months after surgery, and replacement therapy can be safely withdrawn (6, 7).

A distinct subset of patients achieves postoperative serum PTH within the normal range. Yet any reduction or discontinuation of calcium and active vitamin D rapidly lowers serum calcium within days, triggering symptomatic hypocalcemia. Traditionally, ‘hungry bone syndrome’ describes refractory hypocalcemia after parathyroid surgery due to accelerated bone mineralization. Recent studies recognize the same phenomenon in post−thyroidectomy patients with severe preoperative vitamin D deficiency (8–10). The underlying mechanism involves a reparative mineralization phase in the skeleton following prolonged vitamin D deficiency. This phase creates a powerful ‘calcium trap’, driving unidirectional calcium flux from blood into bone. Superimposed on impaired parathyroid reserve, the ability to upregulate PTH-essential for mobilizing bone calcium and enhancing renal reabsorption-becomes compromised. Clinically, hypocalcemia manifests upon calcium withdrawal.

We report a case of a patient who underwent surgery for papillary thyroid carcinoma, whose clinical course fully illustrates the above mechanism: severe vitamin D deficiency was present preoperatively, basal PTH levels remained normal for three years postoperatively, yet multiple attempts to taper calcium supplementation failed, and PTH failed to mount a compensatory rise in response to hypocalcemic stimulation. This case suggests a potential pathophysiological cascade that we hypothesize as a composite of bone hunger and parathyroid reserve insufficiency, though objective confirmation is warranted.

## Case report

A 40-year-old female patient underwent thyroid elastography in July 2022, which revealed multiple bilateral nodules. The largest nodule in the left lobe measured 0.94 cm × 0.87 cm × 0.96 cm, with a vertical orientation, ill-defined margins, and punctate hyperechoic foci. Fine-needle aspiration cytology suggested suspicious papillary thyroid carcinoma, and BRAF V600E mutation testing was positive. Preoperative laboratory evaluation showed serum calcium 2.30 mmol/L, PTH 30.2 pg/mL, and 25−hydroxyvitamin D 12.8 ng/mL, indicating severe vitamin D deficiency. Thyroglobulin antibodies and thyroid peroxidase antibodies were markedly elevated, consistent with concurrent Hashimoto’s thyroiditis. Hemoglobin was 100 g/L and platelet count 500×10^9^/L, serum ferritin, vitamin B12, and folate levels were normal. Other preoperative assessments were unremarkable. (Serum intact parathyroid hormone (iPTH) was measured using a chemiluminescent immunoassay (CLIA) on the Abbott ARCHITECT i2000SR platform (Abbott Laboratories, Abbott Park, IL, USA). The reference range for iPTH in our laboratory is 15.0–87.0 pg/mL. Serum calcium was measured using the arsenazo III colorimetric method (reference range: 2.15-2.55 mmol/L), and 25-hydroxyvitamin D was measured using electrochemiluminescence immunoassay (ECLIA) on the Roche Cobas e601 platform (reference range for sufficiency: ≥30 ng/mL; deficiency: <20 ng/mL; severe deficiency: <12 ng/mL).

In July 2022, the patient underwent total thyroidectomy with bilateral central neck dissection (level VI). The superior and recurrent laryngeal nerves, as well as the bilateral superior and inferior parathyroid glands, were preserved intact. On postoperative day 1, the patient developed mild perioral and acral paresthesia, serum calcium was 2.07 mmol/L, and intravenous calcium gluconate was administered. Symptoms resolved by day 3, with a PTH level of 70.8 pg/mL. The patient was discharged on levothyroxine 75 μg/day. On postoperative day 4, symptoms recurred at home and were relieved by oral calcium carbonate–vitamin D_3_ combination tablets (547 mg elemental calcium and 36.65 μg vitamin D_3_ per tablet, two tablets three times daily). At one month postoperatively, serum PTH had decreased to 3.0 pg/mL, calcium was 2.03 mmol/L, 25−hydroxyvitamin D was 15.85 ng/mL, and TSH was 50.32 μIU/mL. Alfacalcidol 0.5 μg three times daily was added, and levothyroxine was increased to 125 μg/day.

At three months post−surgery, PTH was 26.7 pg/mL and serum calcium 2.20 mmol/L. Calcium and alfacalcidol were discontinued by the physician. One week later, paresthesia recurred, serum calcium was 1.79 mmol/L and PTH 17.4 pg/mL (inappropriately normal given the hypocalcemia). The symptoms resolved after resuming the previous regimen. A second withdrawal attempt at five months led to similar recurrence, after which combination therapy was maintained. Subsequent follow-up at six, 12, and 18 months showed persistently normal PTH levels. At 18 months, alfacalcidol was stopped and calcium was reduced to two tablets three times daily, serum calcium remained normal without symptoms, but calcium could not be fully withdrawn. Multiple attempts to discontinue calcium at 24, 30, and 36 months each induced hypocalcemia within days. As of May 2026, the patient continues oral calcium monotherapy (elemental calcium approximately 1200–1600 mg/day) without active vitamin D, and remains free of nephrocalcinosis or urolithiasis, with normal renal function, serum magnesium, and serum phosphorus.

**Figure 1:**
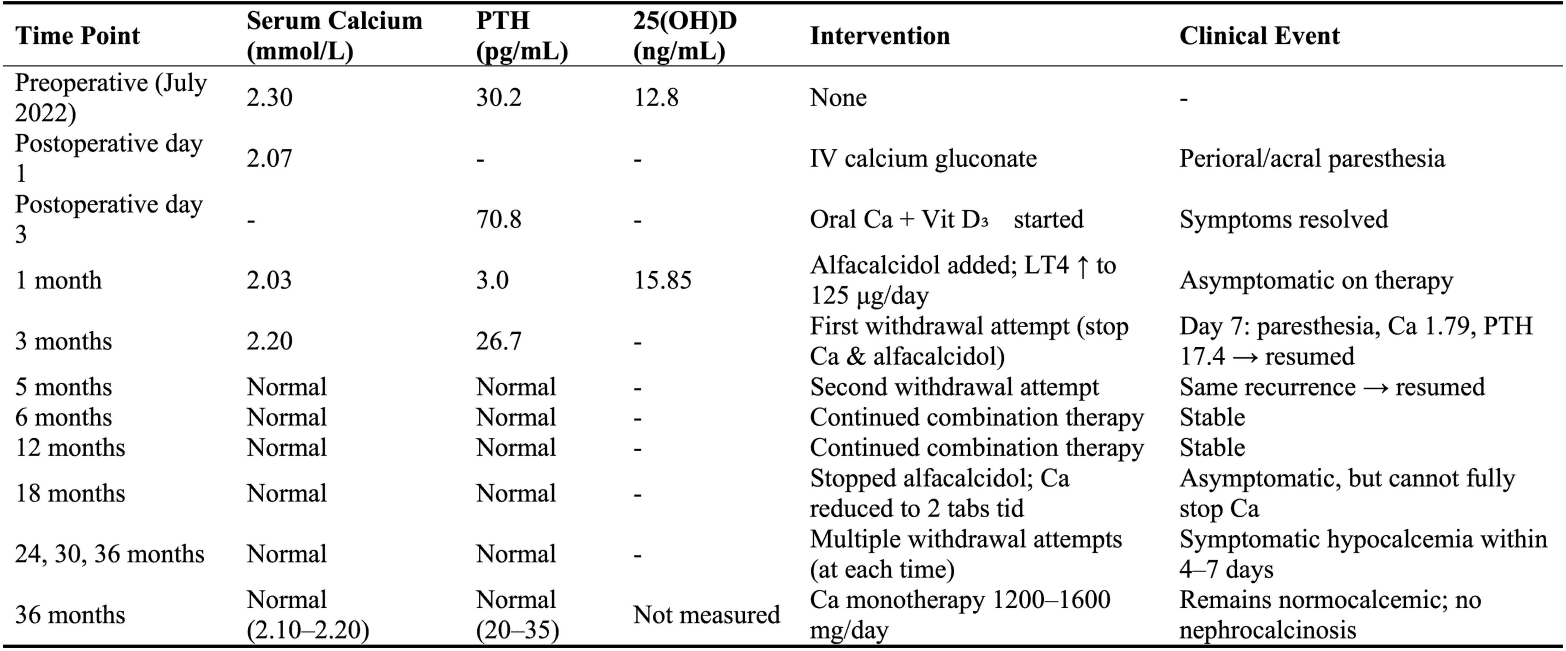
Clinical timeline from preoperative to 36 months post-total thyroidectomy. Key events include preoperative severe vitamin D deficiency, early postoperative hypocalcemia with transient PTH rise, profound PTH drop at 1 month, subsequent normalization of basal PTH, and repeated symptomatic hypocalcemia upon calcium withdrawal without compensatory PTH elevation. Laboratory reference ranges: serum calcium 2.15–2.55 mmol/L, PTH 16–87 pg/mL, 25(OH)D >30 ng/mL. Ca, calcium; PTH, parathyroid hormone; 25(OH)D, 25−hydroxyvitamin D; LT4, levothyroxine; alfacalcidol, active vitamin D analogue; IV, intravenous; tid, three times daily.

A chronological summary of the patient’s clinical course, laboratory data, and treatment modifications is presented in **Figure 1** below.

## Discussion

The unique feature of this case lies in the fact that the patient’s baseline PTH level was completely normal, yet rapid hypocalcemia developed upon calcium withdrawal without a compensatory increase in PTH. This paradoxical observation suggests an underlying functional deficiency masked by a ‘normal’ PTH value. Recent studies have emphasized that the assessment of parathyroid reserve capacity is clinically more valuable than a single absolute PTH measurement (11, 12). In healthy individuals, even mild hypocalcemia induces a several−fold increase in PTH. Conversely, in those with insufficient parathyroid reserve, although basal secretion may be preserved, an amplified response cannot be mounted under stress. Sitges−Serra et al. reported that some patients with normal PTH levels after thyroidectomy still develop hypocalcemia, which is attributable to a functional decline in parathyroid reserve, they also proposed that the PGRIS score, which assesses the number of parathyroid glands preserved *in situ*, better predicts permanent hypoparathyroidism than a single PTH measurement (11). Lee et al. found that a postoperative PTH decline rate exceeding 74.25% independently predicted symptomatic hypocalcemia (OR = 238.4), indicating that the dynamic decline rate reflects parathyroid reserve capacity more accurately than the absolute PTH value (12). In the present case, when serum calcium dropped to 1.79 mmol/L after calcium withdrawal, the PTH level was only 17.4 pg/mL-far below the expected concentration under a hypocalcemic stimulus-confirming this ‘occult’ insufficient reserve.

Mechanistically, the transiently normal PTH level on postoperative day 3 (70.8 pg/mL) followed by a rapid decline to 3.0 pg/mL may reflect a ‘reversible stunning’ of the parathyroid tissue after surgery and subsequent depletion of its functional reserve. Although the surgeon described intact parathyroid gland preservation, subtle damage to the blood supply, secondary ischemia, or postoperative inflammation can all result in a reduction of functional parathyroid mass (3, 13). The patient’s concurrent Hashimoto’s thyroiditis should also be considered: thyroid autoimmunity can be associated with inflammatory infiltration of the parathyroid glands, and case reports have documented the coexistence of Hashimoto’s thyroiditis with hypoparathyroidism (14, 15), potentially further compromising parathyroid functional reserve.

The phenomenon of normal basal PTH with inadequate parathyroid reserve has been increasingly recognized in the literature. Promberger et al. reported a series of eight patients who developed permanent hypocalcemia with normal PTH values after thyroidectomy; the authors proposed that the term “hypoparathyroidism” might be replaced with “parathyroid insufficiency” to better capture this clinical entity (16). Prichard et al. identified that 5% of patients following total thyroidectomy required ongoing calcium supplementation to prevent hypocalcemic symptoms despite a normal serum PTH level, concluding that revascularization of parathyroid cells may be partial, with inadequate parathyroid reserve to avoid symptoms despite measurable PTH levels (17). Furthermore, Anastasiou et al. demonstrated through sodium bicarbonate infusion tests that thyroidectomized patients with normal postoperative calcium and PTH values exhibited a significantly diminished PTH secretory response to acute hypocalcemic stimuli compared with healthy controls (1.77-fold vs. 2.57-fold mean increase, P < 0.001); 6.6% of patients still demonstrated a reduced PTH response at 3 months after surgery (18). Roberts et al. recently reported that despite normalization of PTH levels in 83% of patients with prolonged post-surgical hypoparathyroidism within 14–33 months, 3% of patients had persistent marginally low calcium levels (mean 2.11 mmol/L) in keeping with relative parathyroid insufficiency (19). Collectively, these studies support the concept that a normal basal PTH level does not guarantee adequate parathyroid functional reserve, and that dynamic stress testing is essential for accurate phenotyping.

Intraoperative PTH dynamics also carry predictive value. Evirgen et al. evaluated 88 patients undergoing parathyroidectomy for primary hyperparathyroidism and found that a 15-minute intraoperative PTH decline of ≥75% provided 100% sensitivity and 74.4% specificity for early postoperative hypocalcemia, whereas preoperative alkaline phosphatase (ALP) and parathyroid tumor weight were the strongest independent predictors (20). In the thyroidectomy setting, Moreno Llorente et al., in 742 patients, established that intraoperative PTH declines of ≤62.5% and ≥79.9% optimally predicted transient and high-risk/permanent hypocalcemia, respectively (21). Although these studies were conducted in different surgical contexts, their findings are instructive for our case. First, they reinforce the principle that dynamic PTH changes—whether assessed intraoperatively or through postoperative calcium withdrawal—offer superior predictive value over a single static PTH measurement, aligning with our patient’s experience of normal basal PTH yet failed stress response. Second, the prominent role of preoperative ALP in Evirgen et al.’s cohort highlights a missed opportunity in our case: ALP was not measured preoperatively, precluding a more complete assessment of baseline bone turnover status. Third, the observation that intraoperative PTH decline kinetics effectively stratify hypocalcemia risk provides a framework for understanding our patient’s dramatic PTH fluctuation—from 70.8 pg/mL on postoperative day 3 to 3.0 pg/mL at one month—which likely reflected a substantial intraoperative decline that would have placed her in a high-risk category had monitoring been performed. Collectively, these complementary lines of evidence underscore that PTH dynamics, whether provoked by surgery or by calcium deprivation, are essential for accurate phenotyping of parathyroid functional reserve.

Another key factor contributing to persistent calcium dependence is hungry bone syndrome. Originally described in detail after parathyroidectomy for primary hyperparathyroidism, hungry bone syndrome is characterized by a sudden cessation of longstanding PTH−induced bone resorption, followed by relative predominance of osteoblast activity and massive influx of serum calcium into the skeleton for mineralization (8, 9). Recent studies have shown that severe preoperative vitamin D deficiency can similarly cause an osteoid mineralization defect, resembling an osteomalacic state. Once calcium and active vitamin D are rapidly supplemented after surgery, the bone mineralization process becomes ‘unlocked,’ generating a profound skeletal calcium demand (22, 23). In the present case, the preoperative 25−hydroxyvitamin D level was only 12.8 ng/mL. Although bone turnover markers and bone mineral density were not measured, the presence of severe vitamin D deficiency, together with the rapid development of hypocalcemia upon calcium withdrawal and the lack of an appropriate compensatory PTH response, is consistent with a state of significant bone mineral demand.

Hungry bone syndrome after thyroidectomy, though less commonly reported than after parathyroidectomy, has been documented in various clinical contexts. Guo et al. reported a case of hypocalcemia secondary to hungry bone syndrome after subtotal thyroidectomy for thyrotoxicosis in a 25-year-old woman, highlighting that HBS is due to increased osteoblast-mediated bone formation activity and normal or decreased bone resorption activity (8). Karunakaran et al., in a prospective study of 52 patients undergoing total thyroidectomy for thyrotoxicosis, found that 11.5% of patients developed hungry bone syndrome, and preoperative bone mineral density was significantly lower in those who developed HBS (9). Kusuki and Mizuno described a patient with Graves’ disease who developed severe hungry bone syndrome after near-total thyroidectomy for thyroid storm, with bone formation markers (P1NP) increasing considerably while bone resorption markers (TRACP-5b) decreased, confirming that bone formation exceeded bone resorption postoperatively (10). Gynn et al., in a recent systematic review and meta-analysis of nine studies (n = 2750), demonstrated that preoperative vitamin D supplementation significantly reduced postoperative hypocalcemia (RR: 0.35, 95% CI: 0.18-0.66) and showed trends favoring supplementation for decreasing the risk of hungry bone syndrome (24). These findings collectively support the notion that severe preoperative vitamin D deficiency predisposes patients to a hungry bone state post-thyroidectomy, and that preoperative correction of vitamin D deficiency may mitigate this risk. Future measurement of bone turnover markers (e.g., P1NP and β−CTX) and bone mineral density would be necessary to substantiate this mechanism objectively in our patient.

Regarding the duration of hungry bone syndrome, most cases following parathyroid surgery resolve within several weeks to months, as the accelerated bone mineralization gradually normalizes once adequate calcium and vitamin D are replenished. However, cases of prolonged HBS lasting up to 1–2 years have been documented, particularly in patients with severe preoperative vitamin D deficiency, coexisting osteomalacia, or delayed diagnosis and treatment. Brasier and Nussbaum reported that hypocalcemia after parathyroidectomy could persist for more than 12 months in patients with severe preoperative bone disease (25). Witteveen et al., in a systematic review, described patients with primary hyperparathyroidism and severe vitamin D deficiency who required calcium supplementation for over 18 months postoperatively (26). The exceptionally long duration of calcium dependence in our patient—extending to three years—may be attributable to the combined effect of: 1. ongoing skeletal mineralization demand driven by the repair of profound osteomalacia, and 2. insufficient parathyroid reserve limiting the endogenous PTH surge needed to mobilize bone calcium. This prolonged course underscores the importance of preoperative recognition and correction of vitamin D deficiency to mitigate postoperative calcium disturbances.

We acknowledge that alternative explanations for this presentation exist. First, this case could represent a variant of permanent hypoparathyroidism where the remaining parathyroid tissue is insufficient to meet the demands of a hypocalcemic challenge, despite maintaining normal basal secretion. The inability to mount a PTH stress response is a hallmark of reduced functional reserve. Second, the long-term effects of severe vitamin D deficiency may have reset the patient’s calcium set-point or altered skeletal sensitivity to PTH, leading to a higher calcium requirement to maintain normocalcemia. Third, individual genetic or epigenetic variations in calcium-sensing receptor (CaSR) function or vitamin D receptor (VDR) signaling could modulate the renal and skeletal responses to PTH. While our hypothesis of a composite ‘bone hunger and parathyroid reserve insufficiency’ phenotype offers a unifying explanation, these alternative mechanisms should be considered and investigated in future studies.

With regard to treatment and management, both the 2022 International Guidelines for the Management of Hypoparathyroidism and the 2024 updated European Expert Consensus emphasize that, regardless of the PTH level, patients who are difficult to wean off conventional therapy should be managed as having permanent hypoparathyroidism with long−term replacement therapy, aiming to maintain serum calcium at the lower end of the normal range to avoid hypercalciuria and nephrocalcinosis (27, 28). Cherchir et al. pointed out that chronic hypoparathyroidism requires long−term management as a permanent condition, with maintenance of serum calcium near the lower limit of normal to prevent complications such as nephrocalcinosis and renal insufficiency, after treatment, urinary calcium excretion should be closely monitored to avoid hypercalciuria (29). In the present case, stable serum calcium was achieved with long−term maintenance oral calcium alone, and no hypocalcemia occurred after withdrawal of active vitamin D, suggesting that calcitriol is not mandatory for all patients who are difficult to wean. For patients with a definite diagnosis of hungry bone syndrome, bisphosphonates have been attempted to suppress bone resorption and reduce skeletal calcium demand, however, evidence in the post−thyroidectomy population is limited and potential risks exist, therefore their routine use is not recommended. Shateri et al. reported that among patients receiving preoperative bisphosphonate therapy, the postoperative incidence of hungry bone syndrome (HBS) was as high as 57.9%, and multivariable regression analysis did not demonstrate a significant reduction in HBS risk with bisphosphonate treatment, suggesting that their value in the management of postoperative hungry bone syndrome remains uncertain (30). Corsello et al. described a patient with primary hyperparathyroidism who received preoperative zoledronic acid for severe hypercalcemia and subsequently developed severe and refractory hypocalcemia after surgery, the authors noted that preoperative bisphosphonates might exacerbate the severity of hungry bone syndrome (31). Recombinant human PTH(1−84) could theoretically compensate for insufficient parathyroid reserve (32, 33), but its application in this case was limited by normal baseline PTH levels as well as by cost and accessibility issues. Overall, this patient requires regular monitoring of serum calcium, urinary calcium, and renal function, maintenance of the lowest effective calcium dose, and lifelong follow−up.

The strength of this case report lies in its provision of a three−year continuum of clinical data, including preoperative vitamin D status, postoperative PTH fluctuations, and multiple drug−withdrawal and re−challenge episodes, thereby furnishing a complete chain of clinical evidence for a hypothesized composite pathophysiological model. However, we emphasize that this remains a hypothesis. Limitations include the lack of a standardized intravenous calcium load stimulation test to quantify PTH reserve, the absence of measurements of bone turnover markers and bone mineral density, making the diagnosis of hungry bone syndrome largely based on clinical inference, and the failure to measure 1,25−dihydroxyvitamin D levels, precluding a full assessment of the integrity of the vitamin D metabolic axis. Future studies could prospectively enroll patients with a similar phenotype and employ standardized calcium deprivation tests combined with bone metabolic parameters to clarify the epidemiology and optimal therapeutic strategy for this subtype.

## Conclusion

This case demonstrates that normalization of serum PTH after total thyroidectomy does not signify complete functional recovery of the parathyroid glands. In patients with severe preoperative vitamin D deficiency, significant parathyroid reserve insufficiency may persist even when basal PTH is within the normal range. Once exogenous calcium and active vitamin D are withdrawn, a persistent skeletal calcium demand potentially driven by a hungry bone state can rapidly precipitate symptomatic hypocalcemia. We hypothesize that this clinical picture represents a composite of bone hunger syndrome and parathyroid reserve insufficiency. Clinical practice should therefore emphasize assessment of the PTH stress response, so that an individualized long-term replacement regimen can be formulated for such patients in whom weaning from therapy proves difficult. Further research with objective biomarkers is needed to validate this hypothesis.

## Patient perspective

“Before the surgery, I didn’t even know I had a problem with vitamin D. After the operation, every time my doctor tried to lower my calcium pills or take me off them, within a few days my hands and lips would go numb. It scared me, honestly. I’d feel fine one day, then suddenly my fingers felt like they were buzzing. I went back to the hospital a few times, and they told me my calcium was low. Now I just take my calcium every day. If I forget one dose, the numbness comes back, so I don’t forget. It’s a bit of a hassle, but I’m used to it. I’ve accepted that I might need these pills for the rest of my life. At least I don’t have kidney stones or anything serious. I hope my story helps other patients – maybe they won’t have to go through the same trial and error.”

## Data Availability

The original contributions presented in the study are included in the article/supplementary material. Further inquiries can be directed to the corresponding author.
